# Genetic Variation and Strain Dynamics in Chronic Wasting Disease

**DOI:** 10.3390/v17101308

**Published:** 2025-09-27

**Authors:** Irina Zemlyankina, Melissa Razcon-Echeagaray, Gokhan Yilmaz, Kristen B. Gregg, Sabine Gilch, Samia Hannaoui

**Affiliations:** 1 Calgary Prion Research Unit, Faculty of Veterinary Medicine, University of Calgary, Calgary, AB T2N 1N4, Canada; irina.zemlyankina@ucalgary.ca (I.Z.); melissa.razconecheag@ucalgary.ca (M.R.-E.); gokhan.yilmaz@ucalgary.ca (G.Y.); kristen.gregg@ucalgary.ca (K.B.G.); sgilch@ucalgary.ca (S.G.); 2 Hotchkiss Brain Institute, Cumming School of Medicine, University of Calgary, Calgary, AB T2N 1N4, Canada; 3 Snyder Institute for Chronic Diseases, University of Calgary, Calgary, AB T2N 1N4, Canada

**Keywords:** chronic wasting disease (CWD), prion strains, *Prnp* polymorphisms, CWD strain diversity, host-strain interactions

## Abstract

Chronic wasting disease (CWD) is a prion disease of cervids marked by growing strain diversity and variation in host susceptibility. Central to this complexity are prion protein gene (*Prnp*) polymorphisms, which can modulate pathogenesis by altering the ability of cellular prion protein (PrP^C^) to misfold into infectious prions (PrP^Sc^), or by promoting the emergence of novel strains. Studies in cervids and transgenic rodent models demonstrate that individual polymorphisms influence PrP stability, conversion efficiency, and the selection of PrP^Sc^ conformers, with most variants conferring partial resistance but none offering complete protection. These host–strain interactions define transmission barriers and disease phenotype. Understanding how *Prnp* genotypes shape CWD strain diversity is essential for predicting transmission dynamics, refining surveillance, and assessing zoonotic potential as the disease continues to expand geographically and genetically.

## 1. Introduction

Among prion diseases, infectious and fatal neurodegenerative disorders affecting both humans and animals, chronic wasting disease (CWD) of cervids causes significant concerns among scientists, hunters, farmers, and the general public alike. The disease is caused by infectious prions which consist of PrP^Sc^, the misfolded, β-sheet-rich, and aggregation-prone isoform of the cellular prion protein (PrP^C^) [1]. PrP^Sc^ acts as a template for the conversion of largely α-helical PrP^C^ into a misfolded β-sheet-rich isoform, inducing its structural and conformational change, leading to the accumulation of PrP^Sc^ aggregates. This misfolding process is associated with progressive pathological changes, including neurodegeneration, spongiosis (vacuolar changes), neuronal loss, and reactive gliosis [2,3]. Such extensive damage results in presentation of severe clinical signs; in cervids those signs commonly include excessive salivation, drooping head, difficulty swallowing, ataxia, and severe weight loss or wasting, which earned the disease its name [4].

CWD is characterized by the widespread distribution of PrP^Sc^ throughout multiple tissues in infected cervids, including lymphatic organs, salivary glands, the gastrointestinal tract, skeletal muscles, and blood. Infectious prions have also been identified in bodily fluids and excreta—namely saliva, urine, and feces [5,6,7,8,9,10,11,12,13,14,15,16]. These secretions serve as key routes for environmental contamination. Once released, PrP^Sc^ can bind to environmental substrates such as soil and vegetation, where it remains remarkably stable and infectious for extended periods [17,18,19,20,21,22,23]. This combination of persistent shedding and environmental durability significantly enhances the potential for indirect transmission, contributing to the sustained spread of CWD within both captive and wild cervid populations [5,10,24,25,26,27].

**Figure 1:**
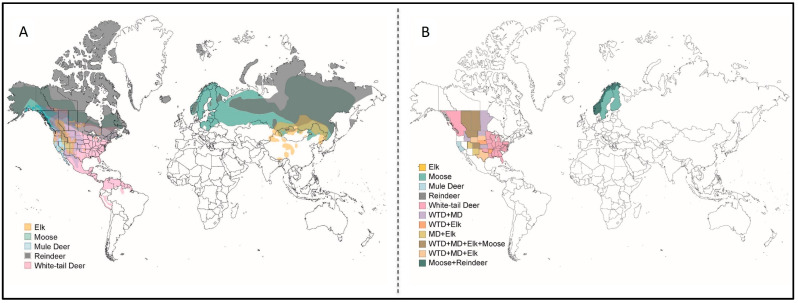
Global distribution of cervid populations and CWD-affected regions highlights potential risk areas for future disease emergence in currently unaffected species. (**A**) Map of the world displaying natural population distributions of cervids that are of interest in CWD research as known affected species (elk, moose, mule deer, white-tailed deer, and reindeer). (**B**) Map of areas of the world of wild cervid species that have confirmed detection of CWD as based on current literature and governmental reports.

CWD is a prion disease that is specific to the Cervidae family which comprises a wide range of wild and farmed animals including but not limited to mule deer (*Odocoileus hemionus*), white-tailed deer (WTD; *Odocoileus virginianus*), and elk (*Cervus elaphus* spp.). The Cervidae family consists of 55 species, many of which can be found in North America, Europe, and Asia alike (Figure 1A).

A key feature that makes CWD particularly concerning is its efficient natural transmission—both vertical and horizontal. Unlike scrapie, which is also highly infectious but largely contained within managed flocks, CWD spreads within and between wild (Figure 1B) and farmed cervids, creating complex transmission dynamics and complicating control efforts at the wildlife–livestock interface [28]. CWD was initially identified in 1967 in a mule deer in a captive facility in Colorado but was formally recognized as a prion disease in the early 1980s [29]. Since then, CWD has spread to 36 U.S. states, 5 Canadian provinces [30], South Korea (imported from Canada) [31], and has also been found in Northern Europe: Norway [32], Finland [33], and Sweden [34]. Among areas affected by CWD in North America, most of them are found East of the Rocky Mountains, generally in the centre-north of the continent.

Although CWD is transmitted efficiently within and among cervid species, prion transmission across species boundaries is generally constrained by so-called transmission barriers [35,36,37,38,39,40,41]. Many of the mechanistic principles discussed below are informed by research from human and rodents, whose PrP sequences differ from cervid prions; nonetheless, these findings remain highly informative for understanding CWD. In this context, “transmission” refers primarily to molecular-level misfolded protein propagation, rather than host-to-host transmission, which is addressed later in this review. A major determinant of these barriers is the degree of amino acid sequence homology between the host’s cellular prion protein (PrP^C^) and the incoming misfolded prion conformer (PrP^Sc^). It governs the efficiency of conformational templating, crucial to pathogenesis, and propagation [38,42,43,44,45]. The “fit” between a given PrP^Sc^ template with its PrP^C^ substrate is not universal across cervid species. It is modulated by the existence of prion strains as well as by genetic differences in the prion protein gene (*Prnp*)—both between species and among individuals within the same species. Variances between substrate and template often reduce the efficiency of PrP^C^ to PrP^Sc^ conversion, thereby impeding cross-species transmission [46,47,48]. In addition to primary sequence compatibility, the cellular environment and the availability of molecular cofactors significantly influence prion replication and host adaptation [49,50,51,52,53,54,55,56]. Experimental data increasingly support the notion that prions do not exist as uniform entities, but rather as a mixture of conformational variants or sub-strains/isolates within a given host. These are commonly referred to as a “quasi-species” or “prion cloud” [57,58,59,60]. It is generally accepted that a prion isolate that preserves both its phenotypic and biochemical characteristics through serial in vivo passages constitutes a distinct strain [61]. Importantly, certain prion strains have demonstrated the capacity to cross species barriers, even posing zoonotic threats. The most notable example is bovine spongiform encephalopathy (BSE), which transmitted to humans, resulting in variant Creutzfeldt–Jakob disease (vCJD) [62,63,64]. The molecular mechanisms underpinning prion adaptation to novel hosts remain incompletely understood. However, several models have been proposed to explain how prions overcome species barriers. One such model proposes the selective amplification of prion conformers that are structurally compatible with the host’s PrP^C^, enabling more efficient conformational templating and propagation [65,66]. Another hypothesis is that incoming PrP^Sc^ can gradually adapt to the host PrP^C^ structure—or vice versa—through conformational shifts that can lead to strain mutations and emergence of novel prion strains [66,67,68]. Recent advancements in high-resolution structural biology, particularly cryo-electron microscopy (cryo-EM), have significantly enhanced our understanding of the conformational basis of prion strain diversity. Cryo-EM has enabled the visualization of prion fibril structures at near-atomic resolution, revealing strain-specific conformations and subtle differences that may underlie transmission efficiency, species tropism, and pathogenicity [69,70]. These structural insights are crucial for identifying molecular determinants of strain behavior and for understanding how prion conformers may evolve or shift when exposed to heterologous PrP^C^ environments. As such, cryo-EM is emerging as a powerful tool in elucidating the molecular basis of prion strain diversity and host adaptation. In support of this, Béringue and colleagues proposed a model in which cross-species transmission may occur through cooperative interactions between subassemblies of prion conformers. This further highlights the complexity of strain dynamics and the role of structural plasticity in zoonotic potential [71,72].

Understanding the intricate interplay between CWD prion strain diversity and host genetic background is critical for assessing transmission dynamics, host susceptibility, and zoonotic potential. The following sections examine *Prnp* polymorphisms across cervid species and their implications for CWD strain evolution, transmission patterns, and potential barriers to cross-species spread.

## 2. Prion Protein Polymorphisms—Modulators of CWD Susceptibility

The *Cervidae* family, the second most diverse lineage within *Ruminantia*, comprises two major subfamilies—*Capreolinae* and *Cervinae*—and includes approximately 55 recognized deer species and subspecies. These taxa vary considerably in morphology, ecology, and behavior [73]. Although the PrP amino acid sequence is generally conserved across cervid species, key polymorphisms have been identified, and these variations appear to influence both host susceptibility and the selection or propagation of specific CWD strains (Table 1). Studies assessing genotype frequencies are still ongoing to better understand CWD propagation in different cervid populations [74,75,76,77].

**Figure 2:**
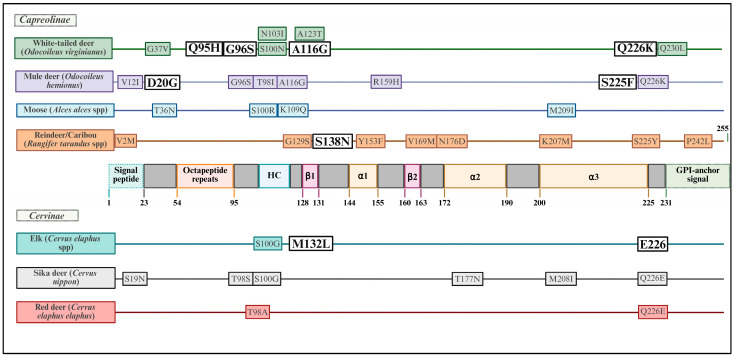
Naturally occurring *Prnp* polymorphisms in cervid species associated with CWD susceptibility and strain dynamics. Cervid species with known natural and/or experimental involvement in CWD transmission are shown alongside key *Prnp* polymorphisms—white-tailed deer (G37V [78]; Q95H [79]; G96S [37]; S100N, N103I, and A123T [80]; A116G [81]; Q226K [82]; Q230L [83]), mule deer (D20G [81]; V21I, G96S, T98I, A116G, and R159H [84]; S225F [85]; Q226K [86]), elk (S100G [87]; M132L [88]; E226 [37]—different from deer Q226 [37]), moose (T36N and K109Q [77]; S100R [89]; M209I [90]), reindeer/caribou (V2M, G129S, S138N, and V169M [91]; N176D (Scandinavian), and S225Y [77]; Y153F, N176D (North America), and P242L [92]; K207M [93]), sika deer (S19N, T98S, T177N, and M208I [94]; S100G and Q226E [95]), and red deer (T98A and Q226E [96]). Single nucleotide polymorphisms (SNPs) are mapped onto a linear schematic of the prion protein (PrP) to illustrate how amino acid substitutions may influence PrP structure and/or function. Codon positions of particular interest are highlighted (**white boxes**), representing sites where genetic variation has been investigated for its potential to confer partial protection or modulate disease dynamics in the context of CWD. HC: hydrophobic core; amino acids mentioned: glycine (G), valine (V), glutamine (Q), histidine (H), serine (S), arginine (R), asparagine (N), isoleucine (I), alanine (A), threonine (T), alanine, lysine (K), leucine (L), aspartic acid (D), phenylalanine (F), methionine (M), tyrosine (T), proline (P), glutamate (E).

A summary of relevant known *Prnp* polymorphisms across cervid species is provided in Figure 2.

## 3. Resistance-Associated Alleles

Naturally occurring amino acid variations in the *Prnp* gene critically modulate susceptibility to prion diseases. The earliest evidence—derived from classical scrapie in sheep—demonstrates strong association between disease outcome and specific codon combinations at positions 136, 154, and 171. These positions typically encode either alanine (A) or valine (V) at 136, arginine (R) or histidine (H) at 154, and glutamine (Q), arginine (R), or histidine (H) at 171. Notably, the V_136_R_154_Q_171_ haplotype is associated with high susceptibility to disease, while A_136_R_154_R_171_ is associated with strong resistance [97,98,99,100]. This molecular understanding catalyzed selective breeding policies aimed to mitigate classical scrapie transmission in livestock populations [98,101,102,103,104]. In humans, the methionine (M) to valine (V) polymorphism at position 129 significantly influences susceptibility to prion diseases such as Kuru and vCJD. Individuals homozygous for methionine (129MM) show increased risk and shorter incubation periods, while heterozygotes (129MV) tend to have prolonged asymptomatic phases. Notably, no vCJD cases have been recorded to date in individuals with the 129VV genotype [105,106,107,108,109].

Within the *Cervidae* family, extensive research has demonstrated that specific polymorphisms in the *Prnp* gene influence susceptibility to CWD, affecting disease incidence, progression, and host–pathogen dynamics. While no naturally occurring variant has conferred complete resistance, several alleles are consistently associated with partial protection or delayed disease onset [28,110,111].

One of the earliest indications of genetic resistance to CWD in elk emerged from the identification of a non-synonymous polymorphism at codon 132 of the *Prnp* gene, which encodes either methionine (M) or leucine (L). The 132M allele is more prevalent and considered the wild-type. This finding drew immediate attention due to its positional equivalence to codon 129 in the human *PRNP* gene. Studies by O’Rourke and colleagues confirmed the presence of the 132ML dimorphism in both free-ranging and farmed elk, and importantly, demonstrated a statistical overrepresentation of 132MM homozygotes among CWD-positive animals compared to CWD-negative controls [88]. These findings suggested a protective effect conferred by the 132L allele, whether in heterozygous (132ML) or homozygous (132LL) form. However, the extent of this protective effect has been debated. A population survey in Colorado by Perucchini and co-workers found that all three genotypes—132MM, ML, and LL—were represented among CWD-infected elk in proportions consistent with their population frequencies, casting doubt on whether the 132L allele alone significantly reduced natural infection risk [112]. Nonetheless, subsequent experimental transmission studies provided stronger evidence supporting its protective role. Hamir and colleagues experimentally challenged elk of known 132 genotypes with pooled CWD-infected brain homogenates—equal parts of 132MM and ML mix—via the oral route. Their studies revealed that 132LL elk exhibited markedly prolonged incubation periods, with delayed onset of clinical signs compared to 132ML, and especially 132MM animals. Although 132LL elk were ultimately susceptible, the incubation period was approximately three-fold longer than in 132MM animals and 1.5 times longer than in 132ML elk, with a deferred pathological pattern, reinforcing the notion that 132L allele imparts partial resistance [113]. Subsequent in vitro analysis using Real-Time Quaking-Induced Conversion assay (RT-QuIC) provided mechanistic insight into these differences. When recombinant PrP substrates containing the 132L variant were used, lag times to amplification were significantly longer than for wild-type (132M) substrates seeded with CWD isolates from 132MM or 132ML elk [114], indicating reduced conversion efficiency.

In WTD, naturally occurring polymorphisms in the *Prnp* gene have been linked to differential susceptibility to CWD. Among these, the most extensively studied polymorphism is at codon 96, which encodes either glycine (G, wild-type) or, less frequently, serine (S), which is found in 23.3% and 30.7% of American and Canadian WTD populations, respectively [115]. Early epidemiological studies in Wisconsin demonstrated that deer with the wild-type genotype were overrepresented among CWD-positive cases, while those carrying at least one 96S allele exhibited reduced infection rates. Experimental investigations have reinforced these findings. In vivo and in vitro studies confirmed that homozygosity for 96S impairs prion propagation and significantly prolongs disease onset in challenged animals, highlighting its protective effect [28,110,111,116]. Oral inoculation experiments, using pooled brain homogenate from two CWD-positive Wisconsin deer (wt/wt genotype), showed that while all inoculated WTD developed clinical CWD, animals with the 96GS genotype had a delayed disease course by approximately nine months compared to wild-type deer [117]. These observations suggest that the 96S variant confers partial resistance by slowing disease progression rather than providing complete protection.

Another notable resistance-associated variant is at codon 95, where glutamine (Q, wild-type) is substituted by histidine (H). Although the 95H allele is rare in wild WTD populations—present in 1.8% and 1.4% of American and Canadian WTD populations, respectively [115]—its presence correlates with a disproportionately low number of CWD cases [75,82,83,115,117,118]. In controlled challenge studies, following oral inoculation with pooled brain homogenate from wt/wt CWD-positive deer, animals carrying 95H—either alone or in combination with 96S—exhibited significantly prolonged incubation periods, with some surviving over 2.5 years post-infection [117]. These deer also showed altered prion distribution patterns, particularly reduced central nervous system (CNS) involvement.

The codon 116 polymorphism, which results in an alanine (A) to glycine (G) substitution (A116G), is another variant of interest. While earlier studies suggested it may not significantly influence susceptibility [75], more recent analyses propose a potential protective role [119,120]. The position of this polymorphism within the central hydrophobic core domain of the PrP^C^—a region essential for prion conversion—supports its biological relevance. It is homologous to codon 113 in the human *PRNP*, where disease-causing mutations have been documented [121,122,123,124,125]. In vitro molecular dynamics simulations revealed that the 116G prion protein variant exhibits increased structural fluctuations, decreased compactness, and signs of conformational instability compared to the wild-type PrP. These changes were most evident in regions surrounding the mutation and resulted in increased β-sheet content, reduced α-helicity, and greater solvent accessibility. The overall destabilization, particularly the enhanced exposure of hydrophobic residues, suggests a conformational mechanistic basis associated with the 116G polymorphism [119].

Finally, a rare polymorphism at codon 226 results in a glutamine (Q) to lysine (K) substitution (Q226K). Although its frequency in the wild population is extremely low—present in only 3.7% of American WTD [115]—studies have shown that individuals carrying the 226K allele are underrepresented among CWD-positive deer [75,82]. In a large-scale genotyping study of over 2000 farmed WTD, including more than 700 CWD-positive animals, Haley and colleagues found that this variant, along with 95H and 116G, conferred a lower relative risk of infection than the more common 96S allele. Risk modeling estimated that the 96G allele was associated with a 2.5-fold greater likelihood of CWD positivity than 96S [115]. Together, these data support the classification of 95H, 96S, 116G, and 226K as resistance-associated *Prnp* alleles in WTD. While none of these variants provide absolute resistance, they collectively contribute to a genotype-dependent gradient of susceptibility and offer valuable insight into host factors that influence CWD pathogenesis.

In mule deer, similar genotype-dependent effects have been observed. Jewell and colleagues studied the polymorphism at codon 225, with a serine (S) to phenylalanine (F) substitution in free-ranging populations across Wyoming and Colorado. In a cohort of 290 CWD-positive deer, only one individual possessed the 225SF genotype. Despite its low frequency, the odds of infection were estimated to be approximately 30-fold lower in 225SF animals compared to those with the wild-type (225SS) genotype. Importantly, 225SF mule deer exhibited delayed onset of severe clinical signs, with median disease progression extending to 36 months post-infection, compared to 23 months post-infection in wild-type individuals [126]. Functional characterization of this polymorphism in vivo was provided by Angers and colleagues, who developed a transgenic mouse model expressing the deer 225F variant, Tg(DeerPrP-F225)5107^+/−^. When challenged with wild-type CWD, these mice showed an incomplete attack rate, with only 2 of 7 developing clinical disease, and those did exhibit substantially prolonged incubation periods (582 and 609 days post infection). Strikingly, when inoculated with CWD prions derived from heterozygous 225SF mule deer, no mice developed disease, despite their susceptibility to wild-type inocula. This suggests that the 225F allele provides strain-specific resistance and does not merely mask infection via heterozygosity. The combination of incomplete penetrance and extended incubation times strongly supports the hypothesis that this polymorphism fundamentally alters prion-host interactions and impairs prion propagation in a strain-dependent manner [39,115,127,128].

The polymorphism at codon 138 of the *Prnp* gene, resulting in a serine (S) to asparagine (N) substitution, has emerged as a key genetic factor modulating resistance to CWD in cervids. This polymorphism has been examined across a range of natural exposure and experimental models [129,130,131,132,133,134,135]. Initial evidence for a protective effect was provided by Rhyan and colleagues, who monitored a captive herd of fallow deer (*Dama dama*; 138NN/226EE wild-type genotype) over seven years in a paddock previously inhabited by CWD-infected mule deer. Despite sustained environmental exposure, none of the 41 fallow deer developed clinical signs of disease, suggesting a degree of natural resistance. Nonetheless, experimental intracerebral inoculation of fallow deer with CWD resulted in disease onset, albeit with delayed incubation periods, limited PrP^Sc^ deposition in the CNS, and no detectable peripheral involvement [131,132], indicating that resistance is route- and strain-dependent rather than absolute. Further insights were gained from studies in reindeer/caribou (*Rangifer tarandus* spp.), which naturally harbor the 138S or 138N allele—paired with 226Q genotype, where 138SS prevalence ranges from 40.2% to 61.4%, 138SN prevalence ranges from 31.8% to 46.0%, and 138NN prevalence ranges from 6.8% to 13.8% [92]. Mitchell and colleagues orally inoculated reindeer (*Rangifer t. tarandus*) with CWD isolates. While 138SS animals developed clinical disease, those with the 138SN genotype showed no signs of disease at 26 months post-inoculation, despite detectable PrP^Sc^ in peripheral lymphoid tissues [129]. This observation suggested a reduction/delay in CNS invasion efficiency in heterozygotes [133]. Moore and co-workers expanded upon this by inoculating reindeer of all three genotypes (SS, SN, and NN) via the intracerebral route. All genotypes developed disease, but co-housing experiments revealed differential peripheral accumulation: naïve 138SN and 138NN animals co-housed with infected reindeer developed detectable prions in spleen and lymph nodes, but not in the brain, suggesting inefficient neuroinvasion under horizontal exposure conditions [134]. To better dissect the mechanisms underlying this polymorphism’s protective effect, the Gilch lab developed gene-targeted mouse models expressing cervid PrP with 138SS, 138SN, or 138NN genotypes [130]. While 138SS mice developed clinical disease upon CWD challenge, 138SN and 138NN mice remained subclinical, with low-level prion seeding activity detected in CNS tissues [130]. Notably, PrP^C^ from 138SN brains served as a poor substrate for CWD amplification using the Protein Misfolding Cyclic Amplification (PMCA) assay, suggesting an altered conformational templating. However, mixing 138SS and 138NN brain homogenates failed to replicate the reduced seeding seen in the brain from 138SN mice, supporting a concept of a cell-intrinsic protective effect resulting from the co-expression of both alleles [130].

Lastly, a study from the Republic of South Korea identified a strong association between a codon 19 polymorphism in sika deer (*Cervus nippon*)—resulting in a serine (S) to asparagine (N) substitution—and susceptibility to CWD [94]. In silico analyses predicted that this substitution may affect the structure and/or function of PrP, potentially contributing to disease alteration [94]. No follow-up studies have been performed to date to further explore the implications on CWD resistance of this polymorphism. A similar signal peptide variant at codon 20, from glycine (G) to aspartic acid (D) has been identified in mule deer, and although less well characterized, it has been associated with potential modulation of CWD susceptibility [83,86].

Research has demonstrated that cervids carrying certain alleles are less likely to test CWD-positive at the time of depopulation, and experience slower disease progression. However, it is important to note that these animals are not entirely resistant to infection.

## 4. CWD Strain Diversity and Their Features

Under controlled conditions, prion strains typically exhibit remarkably stable and heritable phenotypes. For instance, within a given mouse model, the incubation period for a specific strain tends to vary by only a few days, reflecting the high consistency of strain behavior in a defined host background. However, infections with different strains can lead to different disease outcomes [136]. These differences may be evident in incubation time, but also in clinical presentation as was demonstrated by the hyperactivity and lethargy of the hamster-adapted transmissible mink encephalopathy strains “hyper” and “drowsy” [137]. Strains also differ in the pattern and severity of neuropathology including variations in the intensity of vacuolation, and distribution and morphology of PrP^Sc^ deposits in the brain [138,139,140].

At the molecular level, prion strain identity is encoded within the tertiary and quaternary structure of PrP^Sc^. Cryo-EM has revealed that distinct strains adopt characteristic conformations, distinguished by β-sheet architecture, lobe orientation, and fibril organization [69,70,141,142,143,144]. Quaternary features such as aggregate size, solubility, and distribution also shape strain-specific infectivity and may influence disease presentation [145,146,147,148]. These structural characteristics are further supported by biochemical signatures, including protease resistance patterns, glycoform ratios, and conformational stability. Together, these interrelated phenotypic, structural, and biochemical parameters provide a robust framework for distinguishing prion strains, including those of CWD (Table 1), and for investigating how strain diversity influences transmission dynamics, host adaptation, and pathogenesis.

Angers and colleagues were the first to demonstrate the existence of distinct CWD prion strains in both deer and elk, which they designated as CWD1 and CWD2 [149]. Upon first and second passage into transgenic mice overexpressing wild-type deer PrP (Tg(CerPrP)1536), these isolates retained unique strain-specific features, including consistent differences in incubation periods and neuropathological profiles [149]. Notably, elk appeared to support the propagation of more distinct, homogeneous CWD1 or CWD2 strain types, whereas deer were more likely to harbor mixed strain populations, suggesting a broader strain heterogeneity in deer [149]. Subsequent studies using transgenic mice expressing elk PrP [39] revealed that expression of glutamic acid (E) at codon 226—typical of elk—resulted in more phenotypically stable prion strains. In contrast, the glutamine (Q) residue at the same position in deer PrP was associated with greater strain instability and less consistent strain characteristics upon serial passage [39]. This role of host PrP sequence in strain dynamics was further validated using gene-targeted mice expressing either deer (GtQ226) or elk (GtE226) PrP^C^. These models recapitulated the distinct strain selection biases observed in transgenic overexpression systems, confirming the impact of codon 226 on strain stability and propagation [150,151].

**Table 1:** Defining features of the 10 characterized CWD strains. NA (North America), Sc (Scandinavia) White-tailed deer (*Odocoileus virginianus*), elk (*Cervus canadensis*), reindeer (*Rangifer tarandus*), moose (*Alces alces*). WB (Western blot); CGN (cerebellar granular neurons); wt (wild-type); Tg1536 (mice overexpressing wt deer PrP^C^); Tg33 (mice expressing wt deer PrP^C^); Tg60 (mice expressing S96-deer PrP^C^); Tg12 (mice overexpressing 132M elk PrP^C^); TgBOV (mice overexpressing bovine PrP^C^); Cer.Prnp.Wt (gene-targeted mice expressing wt-PrP^C^); Cer.Prnp.138N (gene-targeted mice expressing 138N-PrP^C^); BV (bank vole model); PK (proteinase K). All rodent models listed in this table were inoculated intracerebrally. While not all studies reported high attack rates, each provides valuable insights into prion transmission and host–strain dynamics.

Strain	Region	Species of Origin	PrP^Sc^ Banding Pattern	PrP^Sc^ Stability	PrP^Sc^ Deposition & Neuropathology	Other Features	References
Wisc-1	NA	o Unknown, dominant form in WTD	o 18 to 19 kDa unglycosylated PrP^res^ band	o Compared to 116AG strain: . Higher conformational stability . Higher PK resistance	o Wide and intense, symmetrical PrP^Sc^ deposition (tg(CerPrP)1536)	o Susceptible rodent models include: . Bank voles . Transgenic beaver mouse model . Syrian golden hamsters	[119,120,152,153]
CWD1	NA	o Unknown, identified in elk and deer	o 18 to 19 kDa unglycosylated PrP^res^ band	o Indistinguishable from CWD2	o Continuous and symmetrical PrP^Sc^ deposition o High vacuolation in the hippocampus (Tg(CerPrP)1536)	o Shorter incubation time in Tg(CerPrP)1536 mice than CWD2	[149]
CWD2	NA	o Unknown, identified in elk and deer	o 18 to 19 kDa unglycosylated PrP^res^ band	o Indistinguishable from CWD1	o Asymmetrical distribution of PrP^Sc^ in the inoculated hemisphere o Low vacuolation in the hippocampus (Tg(CerPrP)1536)	o Longer incubation time in Tg(CerPrP)1536 mice than CWD1	[149]
H95+	NA	o Associated with Q95H polymorphism, prevalent in WTD	o 17 kDa unglycosylated PrP^res^ band o High levels of C2 and C3 cleavage without PK treatment o Undetected by 12B2 anti-prion antibody	o Compared to Wisc-1: . Lower PK-resistance	o Localized PrP^Sc^ deposition in brain nuclei (tg60)	o Co-propagates with Wisc-1 in Tg33 mice. o Susceptible rodent models: . Adapts and selected for in Tg60 . Transmissible and pathogenic in C57BL6 mouse model	[152]
LL132	NA	o Elk	o 18 kDa unglycosylated PrP^res^ band	o Compared to 132MM and 132ML: . Higher conformational stability	o Compared to MM and ML (Tg12): . Diffuse neutrophil distribution . Scattered aggregates in the cerebellum . Intense distribution in the CA1 and CA3 hippocampal	o Longer incubation time in Tg12 mice than 132M elk CWD.	[154]
116AG	NA	o Associated with A116G polymorphism, prevalent in WTD	o 18 to 19 kDa unglycosylated PrP^res^ band (classic)	o Compared to Wisc-1 strain: . Low conformational stability . Low PK resistance . In mice and SG hamsters, classic and low bands are observed	o Similar deposition to Wisc-1 o Lower vacuolation in the cortex Tg(CerPrP)1536)	o Lower activity in RT-QuIC, reduced infectivity in CGN (Tg(CerPrP)1536), increased incubation, relative to Wisc-1. o Increased incubation time in Tg60 mice relative to H95+. o No infectivity in bank voles	[119,120,155]
Red deer	Sc	o Red deer	o Main diglycosylated band comparable to BSE o Compared to reindeer: . Lower migration of the non-glycosylated band . Loss of the N-terminal epitope (12B2)	o Not reported	o Deposition in medulla oblongata: . Coarse granular deposits in the neuropil . Perineuronal, intraneuronal, and linear staining (nuclei and axonal tracts)	o Susceptible rodent models include: . TgElk . Bank voles . TgBOV	[33,156,157]
R-NO1	Sc	o Reindeer	o 18 kDa unglycosylated PrP^res^ band (BV) o Absence of 12–13 kDa fragment	o Compared to NA and M-NO1 and M-NO-2 strains: . Lower conformational stability in TgQ226 and GtQ226	o Compared to NA CWD strains (BV), mostly similar, except for: . Strong and diffuse PrP^Sc^ deposition in the visual cortex . Widespread in some cortical layers	o Low infectivity in bank voles o Susceptible rodent models: . Cer.Prnp.Wt mouse model	[151,158,159]
M-NO1	Sc	o Moose	o 18 kDa unglycosylated PrP^res^ band (BV) o Lower amount of 12–13 kDa C-terminal fragment	o Compared to NA and R-NO1 strains: . Higher conformational stability in TgQ226 and GtQ226	o Compared to M-NO2 (BV) . Stronger PrP^Sc^ deposition in the hindbrain	o High infectivity in bank voles o Strong and widespread vacuolation	[151,158]
M-NO2	Sc	o Moose	o 17 kDa unglycosylated PrP^res^ band (BV) o Higher levels of 12–13 kDa C-terminal fragment	o Compared to NA and R-NO1 strains: . Higher conformational stability in TgQ226 and GtQ226	o Compared to M-NO1 (BV): . Absence of vacuolation in the medulla, cerebellum, and hypothalamus	o High infectivity in bank voles o Strong and widespread vacuolation, excluding the hypothalamus	[151,158]
M-NO3	Sc	o Moose	o Comparable to M-NO2 in bank vole model	o Not reported	o Comparable to M-NO2 (BV)	o Susceptible rodent models: . Cer.Prnp.138NN mouse model	[129,158]

A particularly striking example of strain selection linked to host genotype is the emergence of the H95+ strain. Experimental inoculation of Wisc-1—the prototypical CWD strain isolated from wild-type WTD—into WTD carrying the 95H and 96S polymorphisms significantly prolonged incubation periods, often more than doubling the disease course relative to wild-type controls [116,117]. In transgenic mouse models, this delay in disease onset was mirrored [153]. Mice expressing wild-type (95Q/96G) deer PrP (Tg33) propagated Wisc-1 (95Q/96G) efficiently from both wild-type and 96GS CWD sources. However, mice expressing 96S-deer PrP^C^ (Tg60), initially believed to resist CWD infection [160], developed subclinical infections upon exposure to Wisc-1 that subsequently transmitted disease to Tg33 mice, again yielding Wisc-1 [152,153].

In contrast, when WTD-derived CWD isolates encoding the 95H allele (95H/Q or 95H/96S) were passaged into Tg60 mice (96S), they induced clinical disease and selected for a novel strain, H95+, with distinct phenotypic and biochemical properties [152,153,160,161]. This newly emergent strain displayed a prolonged incubation period, altered PrP^Sc^ deposition, unique glycoform profiles, higher conformational stability, lower protease resistance, and differential antibody binding compared to Wisc-1 [152,153]. Remarkably, H95+ prions replicated poorly in Syrian golden hamsters and failed to produce clinical disease yet were fully transmissible and pathogenic in C57BL/6 murine mouse line, further underscoring their strain distinction [152,161]. Conformational stability assays revealed striking similarities between PrP^Sc^ from H95+/Tg60 mice (96S) and that of original 95H/96S deer brains, substantiating that the H95+ strain is a product of Wisc-1 adaptation and selection by the polymorphic allele [152]. This process of host-driven strain emergence highlights the critical role of rare *Prnp* alleles, such as 95H and 96S, in modulating prion strain diversification and stability. Notably, while Wisc-1 and H95+ could co-propagate in Tg33 mice, passage through Tg60 selectively stabilized H95+, confirming the profound strain-modulatory effects of host PrP context. These findings demonstrate that specific *Prnp* polymorphisms not only alter susceptibility and disease kinetics but can also drive the selection and adaptation of distinct prion strains, with potentially broad implications for transmission dynamics, cross-species potential, and strain evolution in the wild.

Another rare polymorphism in WTD occurs at codon 116, where the wild-type allele encodes alanine (A; 116AA). In very rare cases, a glycine variant (G) has been identified [75,162]. This allele is absent from all studied American WTD herds and has been detected in only 2.9% of Canadian prairie populations [115,162]. In an initial investigation examining the impact of the A116G substitution, our group demonstrated that a CWD isolate harboring one polymorphic allele at this position (116A/116G) exhibited lower proteinase K (PK) resistance, reduced conformational stability, lower seeding activity, as well as decreased infectivity in primary cerebellar granular neuron cultures compared to Wisc-1 (116A/116A) [119]. When the 116AG-CWD isolate (95Q/96G/116G) was intracerebrally inoculated into the Tg(CerPrP)1536 mice (95Q/96G/116A), incubation time was prolonged when compared to Wisc-1-CWD isolate (95Q/96G/116A) [119]. Notably, the biochemical properties observed in the isolate were retained upon passaging in these mice. Upon second passage of 116AG prions into the same mouse line, two distinct populations—designated 116AG-short and 116AG-long—were identified based on differential survival times [120]. These phenotypic differences were also reflected at the biochemical level, with 116AG-short exhibiting greater resistance to PK compared to 116AG-long. Furthermore, ten-fold serial dilution of the 116AG and Wisc-1 isolates in Tg(CerPrP)1536 mice revealed distinct vacuolation profiles and lesion scores across the three groups—116AG-short, 116AG-long, and Wisc-1—indicating divergent neuropathological signatures, where unicentric kuru-like plaques in the cerebellum were observed exclusively in mice inoculated with 116AG-long prions. Inoculation of Tg60 mice (S96-deer PrP^C^) with the 116AG isolate confirmed its classification as at least one distinct and novel CWD strain, as evidenced by host susceptibility and a PrP^Sc^ banding pattern distinct from that of H95+ strain—the only CWD strain previously reported to induce prion disease in Tg60 mice—while Wisc-1 fails to transmit in this model [120,152,153]. Interestingly, inoculation of the 116AG-CWD isolate into bank voles—widely regarded as universal prion acceptors due to their permissiveness to a broad range of prion strains—failed to produce clinical disease or detectable PrP^Sc^, despite the fact that bank voles were readily susceptible to the Wisc-1 isolate. This observation was unexpected and contrasts sharply with findings related to the H95+ strain. In the case of H95+, experimental transmission studies in transgenic mice clearly demonstrated that it originated from Wisc-1 through selective pressure imposed by the host PrP genotype [152,153]. Given this precedent, it was anticipated that bank voles inoculated with the 116AG isolate would similarly support the propagation of Wisc-1 or a related strain. The lack of disease transmission in this context suggests that the 116G allele may impose conformational constraints or induce the emergence of a novel, poorly replicating strain that is incompatible with the vole PrP sequence. These results underscore the complexity of prion strain selection and highlight the potential for certain *Prnp* polymorphisms, such as A116G, to modulate prion conformations in ways that disrupt cross-species transmission, even in otherwise permissive hosts [120]. Inoculation of the 116AG isolate into Syrian golden hamsters resulted in an incomplete attack rate, with only one animal developing clinical disease and succumbing to prion infection. However, immunoblot analysis revealed the presence of protease-resistant PrP^Sc^ in the brains of 10 out of 12 inoculated animals. Interestingly, the PrP^Sc^ banding profiles displayed variability in molecular weights, suggesting conformational heterogeneity among the accumulating prion species [120]. These findings support the hypothesis that the 116AG-CWD isolate may contain a mixture of prion strains with differing replication efficiencies and pathogenic potential [120]. The 116AG isolate was further characterized using gene-targeted mouse model encoding deer wild-type (95Q/96G/116A) PrP^C^ (Cer.Prnp.Wt), developed by the Gilch lab, that expresses a physiological level of PrP^C^ [110]. Compared to mice inoculated with Wisc-1, the 116AG group showed reduced PrP^Sc^ load, higher conformational stability, and different quaternary structure profile as shown through sedimentation velocity gradient [155]. Altogether, these results support the existence of a mixture of strains, or at least, a novel 116AG strain within the 116AG-CWD isolate.

To further investigate the molecular mechanisms underpinning the 116G-associated strain characteristics and to unravel the role of the 116G allele in strain selection and propagation, the Gilch lab has developed a gene-targeted mouse model expressing the 116G variant of PrP^C^ (95Q/96G/116G). Ongoing studies using this model are expected to provide deeper insights into how this rare polymorphism influences prion structure, replication dynamics, and host specificity.

The role of the elk polymorphism at codon 132 in modulating CWD susceptibility and prion strain dynamics has been investigated in a stepwise manner through experimental infections bioassays. Early in vivo studies demonstrated that elk homozygous for leucine (132LL), exhibited significantly prolonged incubation periods following oral CWD challenge (pool of 132MM and ML), compared to 132ML and 132MM animals, suggesting a protective effect of the leucine allele at this position [113,163]. Importantly, these findings were further supported by pathological differences in 132 animals, including longer survival, delayed and reduced PrP^Sc^ accumulation, altered neuroanatomical deposition patterns, and increased fibril stability associated with the presence of the 132L allele [114]. These findings were further corroborated in a transgenic mouse model expressing elk PrP variant, the Tg12 mouse line (132M/132M) [154]. In these models, prions derived from 132LL elk retained their strain-specific biochemical signatures, exhibiting longer incubation periods, greater PrP^Sc^ fibril stability, and preserved biochemical and pathological features upon passage in Tg12 mice—indicating the presence of a novel CWD strain associated with the 132LL genotype [154]. These results collectively demonstrate that the presence of the 132L allele not only reduces susceptibility and slows disease progression but also shapes prion strain properties, likely influencing the selection and/or stabilization of divergent CWD strains in elk.

Phenotypic characterization of CWD in European cervids reveals significant strain differences that are not attributable to host *Prnp* polymorphisms. Norwegian moose (*Alces alces* ssp.) CWD is marked by CNS-restricted PrP^Sc^ accumulation, intraneuronal deposits, and a distinct low-molecular-weight profile in immunoblot lacking lymphoid involvement—features not seen in North American moose or Norwegian reindeer CWD [32,159]. Similar presentations have been reported in moose from Sweden and Finland [33]. These consistent pathological features across multiple countries, in animals with conserved PrP sequences, strongly suggest that the observed strain heterogeneity arises independently of known *Prnp* variations. Strain-typing studies in bank voles, and transgenic and gene-targeted mouse models, have further demonstrated that European CWD isolates are distinct from North American strains [151,158]. Subsequent to these studies, we can distinguish one specific CWD strain associated with the Norwegian reindeer (R-NO1), and two unique moose strains (M-NO1 and M-NO2) [151,158]. Recent transmission studies using gene-targeted mice have revealed that CWD isolates from Norwegian moose and red deer (*Cervus elaphus elaphus*) [156] represent distinct prion strains with atypical biological and biochemical features. The Norwegian moose isolate (138SS) was able to overcome the 138N-associated transmission barrier in gene-targeted 138NN mice via intracerebral inoculation, resulting in clinical disease and CNS-restricted PrP^Sc^ accumulation, while failing to replicate in lymphoid tissues even after peripheral exposure [129]. This contrasts with typical North American CWD strains (138S/138S) and indicates a lack of lymphotropism associated with the moose isolate. Biochemical analyses revealed strain heterogeneity, with distinct PrP^Sc^ fragments (~17 vs. ~18 kDa) and differential antibody reactivity, similar to what was observed in the original isolate [159], suggesting potential sub-strain evolution within the host. In contrast, the reindeer isolate (138SS/226EE) replicated efficiently in gene-targeted 138SS mice but showed limited peripheral spread and lymphoid involvement in 138NN animals [129]. In the same study, the Norwegian moose isolate M-NO3 has emerged as a distinct CWD strain with unique biological behavior across host genotypes [129]. Peripheral inoculation of M-NO3 (138SS) into gene-targeted 138NN mice did not result in clinical disease; however, low-level prion seeding activity was detectable in the spinal cord and brain by RT-QuIC. Notably, spleens from all animals were negative for both PrP seeding activity and PrP^Sc^, suggesting that M-NO3 is capable of limited neuroinvasion through peripheral nerves without lymphoid involvement. In contrast, intracerebral inoculation of M-NO3 into gene-targeted 138NN mice produced clinical prion disease in a subset of animals and adapted to the host upon second passage [129]. These findings establish M-NO3 as a CWD prion strain with strain-specific behavior, notably its ability to subclinically persist and ultimately overcome “protective” *Prnp* genotypes compared to North American strains, which typically fail to cause clinical disease in gene-targeted 138NN mice.

Interestingly, the Norwegian red deer isolate also emerged as a distinct strain, capable of inducing clinical disease in TgElk mice, expressing matched genotypes at specific codons (138SS/226EE), but not in 226QQ mice (138SS or 138NN), reinforcing the role of codon 226 in modulating transmission efficiency. Importantly, mice inoculated with red deer isolate displayed minimal lymphoid tropism, consistent with its original host pathology. Further studies by the Torres group also demonstrated that the Norwegian red deer isolate represents a separate strain entity, distinct from both North American CWD, with unique neuroinvasion patterns, host genotype compatibility, and biochemical signatures [157]. Altogether, the growing diversity among Scandinavian CWD strains suggests independent origins—potentially arising from spontaneous events or alternative strain evolution—rather than mere selection driven by *Prnp* polymorphisms.

As a result of these observations, it is clear that genetic differences between both the host and the donor play a crucial role in both developing the disease and potentially evolving existing strains into new ones. Unlike common pathogens, genetics don’t directly affect strains; they act in a more indirect manner.

## 5. Influence of Strains and Host Genetics on Species Barriers

CWD has been the focus of zoonosis studies since 2001 when the first concern over CWD zoonotic potential was studied in three young CJD patients who were known to frequently consume cervid products [164]. This was followed by the first in vitro tests in the following early 2000s and has been extensively studied since then using various primates and humanized mouse models [165,166,167,168,169,170,171,172,173,174], and other in vitro prion replication techniques [35,41,175,176,177,178] in an attempt to determine the likelihood and capacity of cervid prions to seed and elicit disease in humans. So far, there is no clear evidence of the zoonotic capability of CWD like that demonstrated with BSE [62]. Aside from the study examining potential associations in the early 2000s [164], several additional studies have been conducted, yet none have identified a clear link between CWD and the neurological disorders present in the individuals examined [179,180,181,182]. While it is reassuring that so far, no correlation has been drawn between CWD incidence and human dementia cases, it is possible that the criteria for identifying appropriate cases are not fully encompassing.

Every few years, a new CWD strain is discovered, adding a layer of complexity to the issue as it is possible that a zoonotic strain may arise. CWD has an ability to cross species barriers, and different CWD strains show varying infectious capacity and strain presentation even between the species of the same family. For example, gene-targeted mouse models expressing 138NN reindeer PrP demonstrated remarkable resistance to North American CWD strains but readily developed disease from Scandinavian moose CWD [129]. The S96-deer PrP^C^ Tg60 mice—previously thought to be resistant—were infected with the H95+ and 116AG prion strains [120,153]. Bank voles, which are considered universal acceptors, did not develop disease following inoculation with the 116AG strain, and also showed poor transmission efficiency with the Scandinavian reindeer isolate [120,158]. Recently, despite ruling out classical BSE (BSE-C) as the origin, red deer CWD prions displayed biochemical similarities with BSE-C and efficient transmission in TgBov mice, a model predictive of cattle susceptibility [157]. This suggests a concerning potential for interspecies transmission to livestock, especially in regions where cervids and domestic ruminants cohabit. Another recent study demonstrates that a European moose CWD isolate, initially unable to transmit to bovine or human PrP transgenic mice, acquired enhanced host range and zoonotic potential following adaptation in ovine PrP-expressing Tg338 mice [183]. This adaptation enabled subsequent transmission to bovine and humanized mouse models, with codon 129-dependent susceptibility and altered protease-resistant PrP^Sc^ biochemical features distinct from known prion strains [183]. These findings highlight the role intermediate hosts may play in prion adaptation and cross-species transmission. As a result, as more strains are isolated and characterized, one or more may be found to pose a risk to human health.

Although epidemiological studies have yet to confirm transmission of CWD to humans, a growing body of experimental evidence supports the biological plausibility of zoonotic spillover. The species barrier between cervids and humans appears to be influenced by prion strain characteristics, host *Prnp* genotype, and exposure context. Initial studies indicated a strong resistance of humans to CWD; however, recent findings from non-human primate models, transgenic mice overexpressing different variants of human prion protein at codon 129 [167,184], and in vitro amplification techniques like PMCA [178] have prompted a re-evaluation of the potential risk [185]. In non-human primates, squirrel monkeys (*Saimiri sciureus*) have demonstrated susceptibility to CWD through both intracerebral and oral routes, developing progressive neurodegenerative disease with characteristic spongiform changes and PrP^Sc^ deposition [171,172,186]. Conversely, cynomolgus macaques (*Macaca fascicularis*), which are phylogenetically closer to humans, have largely resisted CWD infection [160,172,174]. Evidence from murine models highlights both the robustness of the species barrier and its potential to be overcome. Several studies that used humanized mouse models either overexpressing or physiologically expressing PrP^C^ showed lack of any prion disease hallmarks [165,168,169]. Our group demonstrated that Wisc-1-CWD isolate can induce clinical disease in a 129M-overexpressing mouse model after prolonged incubation, despite minimal classical neuropathology. Unusual protease-resistant PrP^Sc^ low-molecular-weight doublet fragments (12–13 kDa and 7–8 kDa) were detected, divergent from classical prion profiles, although comparable to very rare genetic forms of human prion diseases. This biochemical signature was maintained upon second passage. RT-QuIC revealed widespread prion seeding in the brain, and in feces, suggesting systemic dissemination and shedding. Notably, fecal material transmitted disease to bank voles, restoring a classical protease-resistant PrP^Sc^ profile, highlighting both zoonotic potential and strain adaptability of CWD in humanized models [184]. Barria and colleagues utilized PMCA and demonstrated that such conversion is possible, following some stabilization and adaptation of cervid PrP^Sc^ prior to introduction to human PrP^C^ [35,176,177]. It was also suggested that the physiological environment plays a role in this process, making lymphatic tissue more permissive [187]. The consensus from in vitro studies using North American CWD isolates is that human PrP^C^ is a relatively inefficient, but not completely resistant, substrate for prion conversion [37,178,188]. Scandinavian CWD prions were more efficient at converting prion proteins from other mammals, whereas North American CWD isolates showed a greater tendency to convert human PrP^C^ in vitro [178]. These studies further highlight the threat of the CWD strain diversity and genetic variation to the human–cervid species barrier.

## 6. Conclusions and Perspectives

CWD poses a dynamic and evolving threat at the intersection of wildlife conservation and public health. The expanding diversity of prion strains—shaped by host *Prnp* polymorphisms, environmental pressures, and interspecies interactions—reveals a complex, adaptive system with significant implications for transmission dynamics, disease emergence, and control. Although current epidemiological data remain reassuring, experimental evidence increasingly challenges the presumed impermeability of the cervid-to-human species barrier, with certain CWD strains demonstrating zoonotic potential under permissive conditions. The continued geographic expansion of CWD threatens the sustainability of free-ranging cervid populations, particularly in vulnerable species such as reindeer. While some *Prnp* genotypes offer partial resistance, the demonstrated capacity for strain adaptation suggests these barriers may lessen over time. Although selective breeding for prion-resistant *Prnp* alleles has been effective—or predicted to be effective—in reducing the prevalence of classical scrapie in sheep [189,190,191,192], the widespread occurrence of CWD in free-ranging cervid populations poses a major challenge to implementing similar genetic control strategies, even if resistance-associated alleles would be identified.

Given these limitations, it is increasingly clear that genetic resistance alone will not be sufficient; instead, a deeper understanding of prion strain behavior and host–strain interactions is essential. Current strain typing approaches—largely based on intracerebral inoculation of PrP-overexpressing transgenic mice—are limited in their ability to replicate natural transmission dynamics and selection pressures. Moreover, the absence of standardized strain characterization criteria—including biochemical features—complicates strain classification across studies. Building on this reasoning, recent studies have begun to challenge earlier classifications of CWD strains (e.g., CWD1, CWD2), suggesting a more unified model of North American CWD [151,158,193]. These findings indicate that strain characteristics may be influenced by exposure route and host context, rather than representing entirely distinct strains. For example, Wisc-1 and CWD1, previously considered separate entities, exhibit highly similar biochemical and pathological profiles upon close comparison. This raises the possibility that what has historically been described as multiple strains may, in fact, reflect phenotypic variations within a common conformational circulating strain in a wild-type *Prnp* genetic context. At the same time, emerging evidence from genotype-specific transmission studies challenges the notion of a single dominant strain in North America, and these findings underscore that, despite historical strain convergence, host genotype still plays a critical role in modulating strain phenotype, and not all North American CWD cases can be attributed to a single strain entity.

Hence, future work, using consistent methodologies across studies, should prioritize peripheral and environmental transmission models, investigate the role of host cofactors, and assess strain-specific environmental persistence. A more comprehensive understanding of strain–host interactions will be crucial for translating molecular findings into effective surveillance, mitigation, and public health strategies.

## Data Availability

Not applicable.
